# Visualizing risk modification of hypertensive disorders of pregnancy: development and validation of prediction model for personalized interpregnancy weight management

**DOI:** 10.1038/s41440-024-02024-8

**Published:** 2024-12-11

**Authors:** Sho Tano, Tomomi Kotani, Takafumi Ushida, Seiko Matsuo, Masato Yoshihara, Kenji Imai, Fumie Kinoshita, Yoshinori Moriyama, Masataka Nomoto, Shigeru Yoshida, Mamoru Yamashita, Yasuyuki Kishigami, Hidenori Oguchi, Hiroaki Kajiyama

**Affiliations:** 1 Department of Obstetrics and Gynecology, Nagoya University Graduate School of Medicine, Nagoya, Aichi Japan; 2 Department of Obstetrics, Perinatal Medical Center, TOYOTA Memorial Hospital, Toyota, Aichi Japan; 3 Division of Perinatology, Center for Maternal-Neonatal Care, Nagoya University Hospital, Nagoya, Aichi Japan; 4 Data Science Division, Data Coordinating Center, Department of Advanced Medicine, Nagoya University Hospital, Nagoya, Aichi, Japan; 5 Department of Obstetrics and Gynecology, Fujita Health University School of Medicine, Toyoake, Aichi Japan; 6 Department of Obstetrics and Gynecology, Ogaki Municipal Hospital, Ogaki, Gifu, Japan; 7 Kishokai Medical Corporation, Nagoya, Aichi Japan

**Keywords:** Overweight, Obesity, Pre-conception care, Preeclampsia, Interpregnancy care

## Abstract

The growing recognition of the importance of interpregnancy weight management in reducing hypertensive disorders of pregnancy (HDP) underscores the importance of effective preventive strategies. However, developing effective systems remains a challenge. We aimed to bridge this gap by constructing a prediction model. This study retrospectively analyzed the data of 1746 women who underwent two childbirths across 14 medical facilities, including both tertiary and primary facilities. Data from 2009 to 2019 were used to create a derivation cohort (*n* = 1746). A separate temporal-validation cohort was constructed by adding data between 2020 and 2024 (*n* = 365). Furthermore, the external-validation cohort was constructed using the data from another tertiary center between 2017 and 2023 (*n* = 340). We constructed a prediction model for HDP development in the second pregnancy by applying logistic regression analysis using 5 primary clinical information: maternal age, pre-pregnancy body mass index, and HDP history; and pregnancy interval and weight change velocity between pregnancies. Model performance was assessed across all three cohorts. HDP in the second pregnancy occurred 7.3% in the derivation, 10.1% in the temporal-validation, and 7.9% in the external-validation cohorts. This model demonstrated strong discrimination, with c-statistics of 0.86, 0.88, and 0.86 for the respective cohorts. Precision-recall area under the curve values were 0.90, 0.85, and 0.91, respectively. Calibration showed favorable intercepts (−0.02 to −0.00) and slopes (0.96–1.02) for all cohorts. In conclusion, this externally validated model offers a robust basis for personalized interpregnancy weight management goals for women planning future pregnancies.

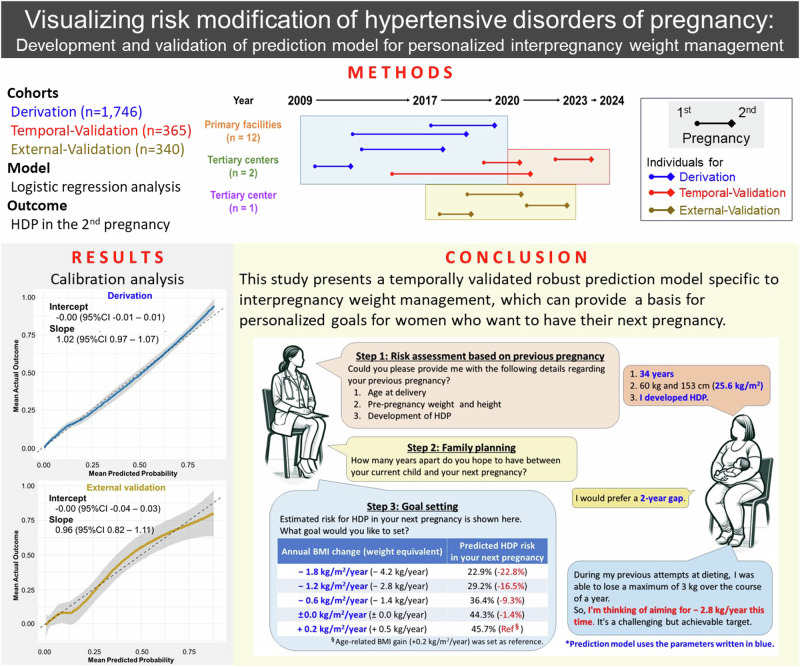

## Introduction

Hypertensive disorders of pregnancy (HDP), with an incidence of 8–10%, is a major cause of maternal mortality, and because there is no effective treatment, prevention is crucial [1]. The implementation of HDP prevention measures can be categorized into two phases: pre- and post-conception. Among post-conception measures, low-dose aspirin is recognized as an effective prophylactic intervention along with gestational weight gain management [2, 3]. Whereas weight control is crucial as a pre-conception measure [4, 5].

Interpregnancy care (IPC) has been endorsed as a pragmatic pre-conception strategy for HDP prevention [4, 5]. The prevailing recommendation for interpregnancy weight management, as a part of IPC, is simply to attain a body mass index (BMI) of 18.5–25.0 kg/m^2^ for prevention of HDP in the subsequent pregnancy [4, 5]. This target may be unattainable for severely obese women who arguably stand to benefit the most from the intervention. Thus, we previously proposed the concept of “annual BMI changes”, defined as the annual pace of pre-pregnancy BMI fluctuations from the previous to the subsequent pregnancy, as an alternative framework allowing for achievable goal setting for all [6–8]. Suppressing annual BMI change below 0.6 kg/m^2^/year was found to minimize the risk “elevation” of HDP development in the subsequent pregnancy [6], which was considered useful for population approach from a preventive medicine perspective [9].

**Fig. 1:**
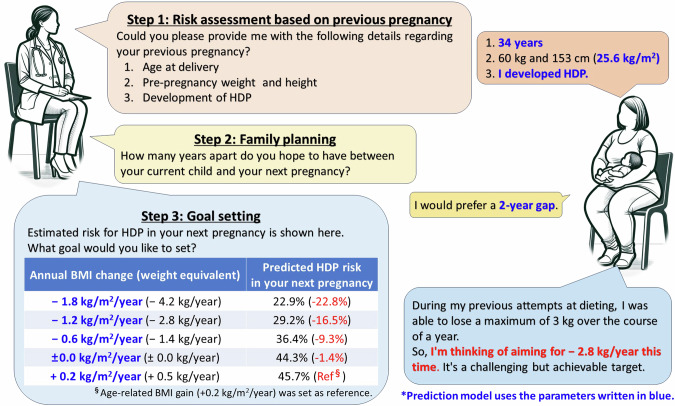
Conceptual Diagram of Bidirectional Communication-Based Interpregnancy Weight Management. Based on the clinical information derived from the risk assessment (step 1) and family planning (step 2), the prediction model estimated the risk of HDP in the next pregnancy corresponding to annual BMI changes (step 3). BMI body mass index, HDP hypertensive disorders of pregnancy

As for high-risk approach aiming for risk “reduction”, precision medicine is considered ideal [9, 10], because weight reduction requires their own active involvement [11]. It is known that promoting patient behavior change and posits that behavior modification is facilitated by understanding one’s own risk, and anticipated outcomes of action or inaction [12]. With using a prediction model, visualizing the current risk and the degree of risk modification can be achieved through their own efforts. As shown in Fig. 1, our proposing bidirectional communication-based prevention strategy initiates with a risk assessment, evaluating parameters from the previous pregnancy (Step 1: risk assessment). Subsequently, the model inputs the period until the next pregnancy and sets a timeframe for weight management (Step 2: family planning). Finally, prediction model utilizes these parameters to visually represent how modifications in annual BMI change can alter the probability of HDP risk in future pregnancies, with the age-related BMI gain (0.2 kg/m²/year) [11, 13, 14] serving as a reference (Step 3: goal setting). A suitable prediction model is required to implement this strategy, but currently not available.

Previously, some prediction models for HDP have been proposed, but they were all designed to evaluate and intervene during the post-conception phase [15, 16]. Notably, no models are specifically aimed at pre-conception measures, including inter-conception weight management. Thus, the objective of this study was to develop and validate a prediction model for interpregnancy weight management aimed at reducing the risk of HDP in future pregnancies.

Point of view

- **Clinical relevance**
  This robust prediction model assists postpartum women by offering clear insight into their risk of hypertensive disorders of pregnancy (HDP) in their future pregnancy. It enables them to set interpregnancy weight management goals, potentially reducing the risk of HDP.
- **Future direction**
  A prospective study, including interventional trials, is required to establish an effective management protocol based on this prediction model.
- **Consideration for the Asian population**
  Given the differences in the relationship between BMI and health risks in Asian and European populations, developing models tailored to Asian populations, like this one, is crucial.

## Methods

### Derivation and validation Cohort

**Fig. 2:**
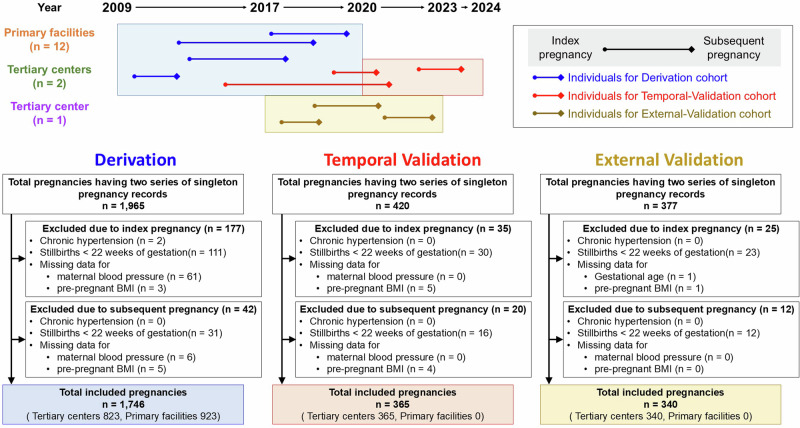
Flowchart Showing the Composition of the Derivation, Temoral- and External-Validation Cohorts. This image presents a schematic outline of the selection of the study cohorts. Each case in the study is depicted by a sequence of symbols: a circle (●) for the initial or index pregnancy and a diamond (◆) for the subsequent pregnancy. The derivation cohort comprised cases from 12 primary facilities and 2 tertiary centers with subsequent pregnancies that resulted in deliveries between 2009 and 2019, represented by blue lines. The temporal-validation cohort consisted of cases exclusively from 2 tertiary centers with subsequent pregnancies delivered between 2020 and 2024, as shown by the red lines. The external-validation cohort consisted of cases from another tertiary center with subsequent pregnancies that resulted in deliveries between 2017 and 2023, as shown by the yellow lines. BMI body mass index

We used the dataset from our previous study [6] as the derivation cohort. This dataset consisted of the electronic medical records of women aged ≥15 years with singleton pregnancies who had two deliveries at 2 tertiary facilities (Nagoya University Hospital and TOYOTA Memorial Hospital, Aichi Prefecture) and 12 private obstetric facilities (Kishokai Medical Corporation, Aichi and Gifu Prefecture) between 2009 and 2019 (Fig. 2). We defined the first and second pregnancies during the study period as the index and subsequent pregnancies, respectively. The exclusion criteria were stillbirth before 22 weeks of gestation, chronic hypertension, and missing data for maternal blood pressure and pre-pregnancy BMI. Using the same inclusion and exclusion criteria, data from the two tertiary centers between January 2020 and June 2024 were retrospectively added. Women with singleton pregnancies who delivered a subsequent pregnancy during this period were included in the temporal-validation cohort. There were no overlapping individuals in both cohorts: they were independent. Furthermore, the external-validation cohort was constructed using data from another tertiary center, Ogaki municipal hospital in Gifu prefecture, covering the period from January 2017 to December 2023.

This study was approved by the ethics board of Nagoya University Hospital (approval number: 2015–0415), and the study was conducted following the Declaration of Helsinki. The requirement for informed consent was waived because of the retrospective nature of the study. We followed the Transparent Reporting of a Multivariable Prediction Model for Individual Prognosis or Diagnosis (TRIPOD) guidelines.

### Outcome

The primary outcome was the development of HDP during the subsequent pregnancy. Our primary outcome, HDP, comprised gestational hypertension and preeclampsia, that is, the development of hypertension after 20 weeks of gestation. HDP was reassessed by two obstetric specialist (ST) and (MN) who reviewed the medical records of all participants with using the same current diagnostic criteria [17]. Although chronic hypertension is a subtype of HDP [17], because the primary outcome was the onset of the HDP, it was placed in the exclusion criteria.

### Definitions of variables

We used self-reported maternal pre-pregnancy body weight obtained during routine practice and calculated the pre-pregnancy BMI (kg/m^2^). ΔBMI was defined as a change in pre-pregnancy BMI from the index pregnancy to the subsequent pregnancy, as previously reported [18, 19]. The pregnancy interval was defined as the interval from the expected date of delivery (EDD) of the index pregnancy to that of the subsequent pregnancy. EDD was determined based on the last menstrual period or measurement of the crown-rump length during routine practice. The annual BMI change (kg/m^2^/year) was calculated as the ΔBMI/pregnancy interval [6–8].

### Developing a prediction model

Using data from the derivation cohort, we constructed a prediction model for the probability of HDP during subsequent pregnancies (predicted probability). This model was calculated using a logistic regression equation [20] with our previously reported optimal five predictors: maternal age and pre-pregnancy BMI at the index pregnancy (Age and BMI, respectively), presence (1) or absence (0) of HDP at the index pregnancy (HDP^ind^), pregnancy interval (Pi), and annual BMI change (ABc) [6].

$${Predicted\; probability}=\frac{1}{1+{e}^{-Y}}$$

$$Y = 	 \; 0.045\times {Age}+0.188\times {BMI}+2.580\times {{HDP}}^{{ind}}\\ 	 +0.319\times {Pi}+0.634\times {ABc}-9.759$$

### Statistical analysis

The differences in characteristics among the three cohorts were statistically analyzed using one-way analysis of variance (ANOVA). Dunnett’s test post hoc ANOVA test was used to identify significant differences between derivation and two validation cohorts. Chi-square tests were used for categorical variables. Statistical significance was set at two-tailed *p*-value of <0.05.

Model performance was evaluated across all three cohorts with respect to discrimination, calibration, and clinical usefulness. Discrimination was evaluated by estimating the area under the receiver operating characteristic curve (c-statistic) [21] and performing precision-recall curve analysis [22]. Calibration was evaluated by plotting flexible calibration curves based on locally estimated scatterplot smoothing (LOESS) [23]. The clinical usefulness of the model was evaluated using decision curve analysis, which determines the net benefit across a range of threshold probabilities [24]. The net benefit was calculated as the number of true positives (TPs) minus the number of false positives (FPs), with the latter weighted according to the threshold probability. Plots were generated to compare the net benefit of the three scenarios: one assuming all patients were at high risk, another assuming no patients were at high risk, and one based on the logistic regression prediction model [24]. Statistical analyses were performed using SPSS ver. 28.0 (IBM, Inc.) and R ver.4.1.3 (R Foundation for Statistical Computing).

## Results

### Baseline characteristics and outcomes of derivation and validation Cohorts

The derivation, temporal-validation, and external-validation cohorts included 1746, 365, and 340 individuals, respectively (Table 1). No data regarding outcomes or predictors were missing. HDP in the subsequent pregnancy occurred 7.3% (128/1,746) in the derivation, 10.1% (37/365) in the temporal-validation, and 7.9% (27/340) in the external-validation cohorts. Concerning the index pregnancy parameters, pre-pregnancy BMI was higher in both the temporal-validation (22.1 ± 4.0 kg/m²) and external-validation cohorts (22.3 ± 4.8 kg/m²) compared to the derivation cohort (20.9 ± 3.3 kg/m²) (*p* < 0.001). There were also significant differences in the prevalence of overweight ( ≥ 23.0 kg/m²) individuals, with the highest prevalence in the temporal-validation cohort (41.1%), followed by the external-validation cohort (34.1%) and the derivation cohort (16.8%) (*p* < 0.001). Regarding the interpregnancy parameters, the pregnancy interval was significantly longer in the temporal-validation (3.2 ± 2.2 years) and external-validation cohorts (2.5 ± 1.1 years) compared to the derivation cohort (2.3 ± 0.9 years) (*p* < 0.001). In the subsequent pregnancy parameters, the rate of HDP in the subsequent pregnancy did not differ significantly among the three cohorts (*p* = 0.192).

**Table 1:** Comparison of the characteristics between the derivation and validation cohorts

	Derivation (*n* = 1746)	Temporal-Validation (*n* = 365)	External-Validation (*n* = 340)	*p*-value
Index pregnancy				
Age, years	30.5 ± 4.8	30.6 ± 5.1	29.2 ± 5.1^b^	<0.001^a^
Pre-pregnancy BMI, kg/m^2^	20.9 ± 3.3	22.1 ± 4.0^b^	22.3 ± 4.8^b^	<0.001^a^
Overweight (≥23.0 kg/m^2^)	294 (16.8)	150 (41.1)	116 (34.1)	<0.001^a^
Primiparity	1207 (69.1)	243 (66.6)	247 (72.6)	<0.001^c^
HDP	202 (11.6)	51 (14.0)	32 (9.4)	0.167
GA at delivery, weeks	39.1 ± 2.1	37.9 ± 3.7^b^	38.3 ± 3.0^b^	<0.001^a^
Birthweight, g	2976 ± 498	2751 ± 724^b^	2853 ± 641^b^	<0.001^a^
Neonatal sex, male	942 (54.0)	158 (43.3)	176 (51.8)	0.001^c^
Interpregnancy period				
Pregnancy interval, years	2.3 ± 0.9	3.2 ± 2.2^b^	2.5 ± 1.1^b^	<0.001^a^
ΔBMI, kg/m^2^	0.44 ± 1.43	0.80 ± 2.16^b^	0.50 ± 1.89	<0.001^a^
Annual BMI change, kg/m^2^/year	0.22 ± 0.84	0.29 ± 0.86	0.25 ± 1.04	0.312
Subsequent pregnancy				
HDP	128 (7.3)	37 (10.1)	27 (7.9)	0.192
GA at delivery, weeks	39.0 ± 1.5	38.2 ± 3.7^b^	38.2 ± 2.6^b^	<0.001^a^
Birthweight, g	3052 ± 422	2917 ± 682^b^	2934 ± 547^b^	<0.001^a^
Neonatal sex, male	893 (51.2)	185 (50.7)	173 (51.0)	0.889

Data are presented as mean ± standard deviation or *n* (%)

^**a**^Statistically significant by one-way ANOVA

^**b**^Statistically significant comparing to Derivation cohort by one-way ANOVA with post-hoc Dunnett’s t-test

^**c**^Statistically significant by chi-square test

*BMI* body mass index, *HDP* hypertensive disorders of pregnancy, *GA* gestational age

### Model performance

**Fig. 3:**
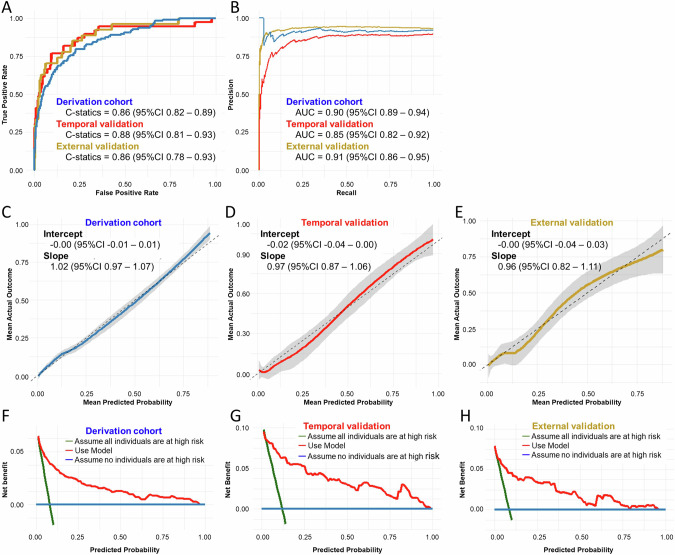
Model Performance. **A** Receiver Operating Characteristic (ROC) Curves. The graph presents the ROC curves for the derivation cohort (blue) and temporal- (red) and external- (yellow) validation cohorts; the *x*-axis indicates the false-positive rate (FPR), and the *y*-axis shows the true-positive rate (TPR). ROC, receiver operating characteristic; 95% CI, 95% confidence interval. **B** Precision-Recall Curves for the Derivation and Validation Cohorts. The plot delineates the precision-recall relationship for the derivation cohort (blue) and temporal- (red) and external- (yellow) validation cohorts. The *x*-axis represents recall, and the y-axis indicates precision. **C**–**E** Calibration Analyses. These plots illustrate the calibration of the prediction model for the derivation (**C**) and temporal- (**D**) external- (**E**) and validation cohorts, by juxtaposing the mean predicted probability (*x*-axis) with the mean actual outcome (*y*-axis). The loess-smoothed line represents the nonlinear relationship between these two variables, and the shaded region indicates the 95% confidence interval (CI) around the smoothed estimates. An ideal prediction model would have all its data points, and the smoothed line would adhere closely to the dashed 45-degree reference line, indicating signaling-impeccable calibration. **F**–**H** Decision Curve Analyses. Decision curve analysis for the derivation (E), temporal- (**F**), and external- (**H**) validation cohorts. These graphs compare the net benefits derived from the three risk-stratification strategies across a range of threshold probabilities. The red line indicates the net benefit of using our predictive model, the green line assumes that all individuals are at high risk, and the blue line indicates that no individual is at high risk

The c-statistics for the derivation, temporal-validation, and external-validation cohorts were 0.86 (95%CI 0.82–0.89), 0.88 (95%CI 0.81–0.93), and 0.86 (95%CI 0.78–0.93), respectively (Fig. 3A). Precision recall analyses also showed that the area under the curve was 0.90 (95%CI 0.89–0.94), 0.85 (95%CI 0.82–0.92), and 0.91 (95%CI 0.86–0.95) for the respective cohorts. (Fig. 3B). These results indicate that the individuals in each cohort could be strongly discriminated by the prediction model. Calibration analyses revealed high slopes and low intercepts (Fig. 3C–E). Specifically, the derivation cohort exhibited an intercept of −0.00 (95%CI −0.01–0.01) and a slope of 1.02 (95%CI 0.97–1.07). Similarly, the temporal- and external-validation cohorts presented intercepts of −0.02 (95%CI −0.04–0.00) with a slopes of 0.97 (95%CI 0.87–1.06), and −0.00 (95%CI −0.04–0.013) with a slopes of 0.96 (95%CI 0.82–1.11), respectively. Notably, the ideal line (dashed line) fell within the 95%CI of the all calibration curves, indicating a well-calibrated model.

### Evaluation of the clinical usefulness and application of the model

Decision curve analysis for the three cohorts (Fig. 3F–H) demonstrated that the ‘Use model’ (red line) consistently surpasses the ‘Assume all individuals are high risk’ (green line) and ‘Assume no individuals are high risk’ (blue line) across the threshold probabilities. This indicates that our model provides a greater net benefit compared to the extreme strategies of treating everyone or treating no one.

## Discussion

### Main findings

Our study successfully constructed and validated a logistic regression-based interpregnancy weight management-focused prediction model that exhibited high accuracy in forecasting HDP in subsequent pregnancies. The existing prediction models are designed for assessment at the post-conception stage [15, 16, 25], and this is the first prediction model for interpregnancy weight management, a type of pre-conception care. Unlike typical supervised learning assumptions that expect similar characteristics across derivation and validation cohorts [26], our model maintained reliability even with divergent cohort characteristics, underscoring its robustness and generality. Remarkably, this highly accurate prediction model comprises a simplified set of five key variables, which greatly enhances its practicality in clinical use.

### Interpretation

“Interpretability” and “Accuracy” are important in constructing a prediction model [27]. “Interpretability” is a passive property of a model, which indicates the degree to which a given model can be interpreted by a human observer. “Accuracy” refers to the degree of the model performance. Interpretability and accuracy generally have a trade-off relationship [28]. Logistic regression, a conventional method, has higher interpretability and lower accuracy, whereas machine learning has lower interpretability and higher accuracy [27].

It is widely recognized that individuals are reluctant to adopt tools that cannot be directly interpreted. Thus, there are concerns about the abundance of research that emphasizes new algorithms without focusing on user-friendliness, practical interpretability, or efficacy for end users [29, 30]. Furthermore, when implementing machine learning-based prediction models in clinical settings, the operational environment of programming languages used in model construction, such as R or Python, is necessary. In contrast, prediction models developed using logistic regression analysis can be replicated using any software capable of logarithmic calculations. Therefore, the results of the present study, obtained with reasonable accuracy using logistic regression analysis, are advantageous for clinical applications.

A recent meta-analysis on externally validated prediction models for HDP concluded that models based solely on maternal information exhibit equivalent predictive accuracy to those augmented with various biomarkers [25]. This suggests that maternal information plays a significantly crucial role in predicting the occurrence of HDP. Consequently, the high predictive accuracy achieved in our study using only clinical data aligns logically with these insights. This capability indicates the potential for high-accuracy predictions in low-resource countries and regions.

### Implications

Our prediction model makes women aware of their own HDP risks before pregnancy and visualize that one can reduce risk through one’s efforts. Using the outputs, establishing weight management goals through bidirectional communication between healthcare providers and women is beneficial for the implementation of interpregnancy weight management. This interactive process allows the integration of each individual’s risk tolerance and commitment to lifestyle modifications, thereby enabling the setting of personalized and realistic targets. That is, bidirectional communication enables women to consider their past experiences and current environment to ascertain the degree of attainable or appropriate weight loss. Concurrently, healthcare providers can evaluate whether the goals are reasonable.

### Strength and limitations

The primary strength of this study is its novel approach, which focuses on pragmatic applications for interpregnancy weight management, a part of IPC. Secondary, the prediction model, which sets weight management objectives based on annual BMI changes, ensures consistency and reliability irrespective of variations in pregnancy intervals. Additionally, the model design, utilizing only five optimal covariates, not only enhances user-friendliness but also reduces the risk of inaccuracy due to missing data in clinical use.

This study had some limitations. First, it was retrospective, and the annual BMI changes were not intervention-induced. Further studies are required to determine whether intervention-induced weight reduction reduces the risk of HDP in subsequent pregnancies. Accordingly, we plan to conduct a prospective study on weight management as a part of IPC using the prediction model developed in this study. Second, self-reported body weight was used to calculate the pre-pregnancy BMI. However, as almost all participants weighed themselves during antenatal checkups in the first trimester of pregnancy, the difference between their self-reported and actual weights was likely to be minimal. Third, all validations in this study were conducted using pregnancy data from within Japan. To guarantee its generalizability, geographic validation is necessary. Nevertheless, the prediction model developed herein utilizes solely clinical information, making it applicable across countries and regions regardless of their economic status.

### Perspective of Asia

The global rise in obesity is a growing issue, and Asia is no exception. In particular, Asians are more sensitive to the health risks of obesity compared to Western populations [31]. However, a major problem is that no standardized methods for weight management during IPC have been established [32]. Additionally, cultural, racial, and economic factors have been reported to influence postpartum weight loss programs [32, 33].

This study succeeded in developing a prediction model for setting weight management goals during IPC, using a cohort predominantly composed of Asians. We believe this represents a significant step towards more personalized medicine, in contrast to the uniform goal-setting recommended by conventional guidelines [4, 5]. Moving forward, we plan to conduct further research to establish a practical management system based on this prediction model in Asian population. Our future research will focus on how effective management can improve perinatal outcomes and, ultimately, extend healthy life expectancy.

## Conclusion

This interpregnancy weight management-focused prediction model is anticipated to offer personalized risk estimations and a basis for establishing weight management goals intended to decrease the risk of future HDP for women who want to have their next pregnancy.

## Supplementary information

Supplementary information
